# A Case Report of Giant Posterior Cerebral Artery Aneurysm

**DOI:** 10.7759/cureus.48336

**Published:** 2023-11-05

**Authors:** Abdulaziz A Albalawi, Mohammed A Alghamdi, Samah F Alkuraydis, Basim K Alraddadi, Sharif M Alfeki

**Affiliations:** 1 General Practice, University of Tabuk, Tabuk, SAU; 2 General Practice, Qassim University, Buraydah, SAU; 3 General Practice, King Abdulaziz University, Jeddah, SAU; 4 Emergency Medicine, Dallah Hospital, Riyadh, SAU

**Keywords:** case report, magnetic resonance imaging, headache, aneurysm, posterior cerebral artery

## Abstract

Cerebral aneurysms, characterized by localized arterial dilations, represent a significant neurological concern, often remaining asymptomatic until a critical event triggers clinical manifestation. We present the case of a 64-year-old male who presented with a severe headache and visual disturbances. The diagnostic workup revealed significant dilatation of the right posterior cerebral artery, confirmed as a large aneurysm through magnetic resonance angiography. Endovascular coiling was chosen as the primary management option, resulting in a successful procedure and near-complete resolution of symptoms. This case report underscores the clinical significance of posterior cerebral artery aneurysms, a relatively rare yet potentially life-threatening vascular anomaly.

## Introduction

Cerebral aneurysms, localized dilations of cerebral arteries, are a critical neurological condition that can remain asymptomatic until a critical event triggers clinical manifestation. These vascular abnormalities affect approximately 2-5% of the general population and are most commonly observed in individuals aged 40 to 60, often with a slight female predilection [1]. Hypertension, smoking, and genetic predisposition are known risk factors for aneurysm development. Ruptured aneurysms can lead to subarachnoid hemorrhage, highlighting the significance of early detection and intervention [1].

The pathophysiology of cerebral aneurysms involves the degradation of the arterial wall's structural components, particularly the tunica media, resulting in a weakened vascular structure. The risk of rupture is significantly influenced by factors such as aneurysm size, location, and the presence of accompanying symptoms [2]. This case report centers on a 64-year-old male who sought medical attention with an acute and severe headache accompanied by visual disturbances. Furthermore, the case highlights the imperative role of a multidisciplinary approach, bringing together the expertise of neurologists, radiologists, and neurosurgeons, to ensure well-informed decisions in the management of these potentially life-threatening vascular abnormalities.

## Case presentation

A 64-year-old male presented to the emergency room with a chief complaint of a severe headache and visual disturbances. The patient described the headache as sudden in onset, with an intensity that he rated as 9 out of 10 on a pain scale, where 0 represents no pain and 10 represents the worst pain imaginable. He characterized the headache as throbbing and localized to the right side of his head. The headache was not alleviated by changes in position or rest and was not associated with any specific relieving factors.

In addition to the severe headache, the patient reported experiencing visual disturbances, including blurry vision and transient flashes of light in the right visual field. These visual disturbances were persistent and not relieved by any specific actions or medications. These symptoms had been progressively worsening over the past week. The patient also mentioned experiencing occasional nausea, which he described as mild and transient.

The patient had a history of hypertension and type 2 diabetes, but these conditions were well-controlled with medications. The patient had no history of seizures. There were no other significant symptoms reported at the time of presentation.

In the review of systems, there were no reports of trauma, fever, loss of consciousness, or focal neurological deficits other than visual disturbances. There was no history of recent infections, anticoagulant use, or a family history of cerebrovascular diseases.

On examination, the patient was in mild distress due to the ongoing headache. Vital signs, including blood pressure, heart rate, and respiratory rate, were within normal limits. The neurological examination was notable for a right homonymous hemianopia, which was confirmed during confrontation testing. Other cranial nerve examinations were unremarkable. There were no focal motor or sensory deficits, and the patient's gait and coordination were normal.

Initial laboratory investigations, including complete blood count, electrolytes, renal and liver function tests, and coagulation profiles, all fell within normal reference ranges. Notably, the patient's inflammatory markers, including erythrocyte sedimentation rate and C-reactive protein, were within normal range, ruling out infectious or inflammatory etiologies.

**Figure 1:**
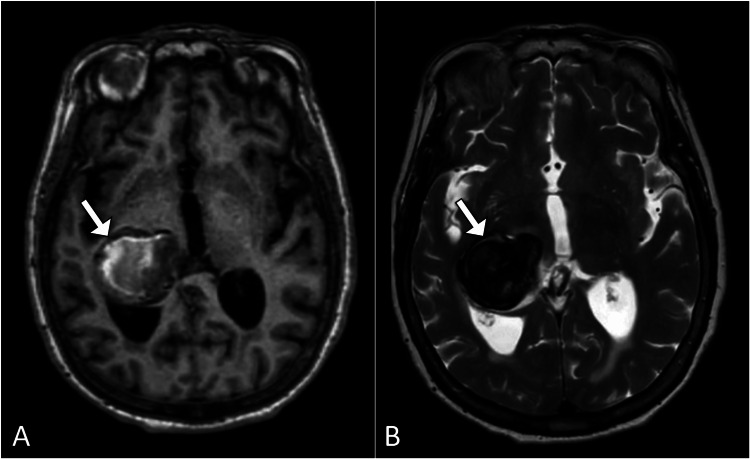
Axial brain magnetic resonance images (A) T1-weighted and (B) T2-weighted displaying an extra-axial hemorrhage (denoted by white arrows) located adjacent to the occipital horn of the right lateral ventricle.

**Figure 2:**
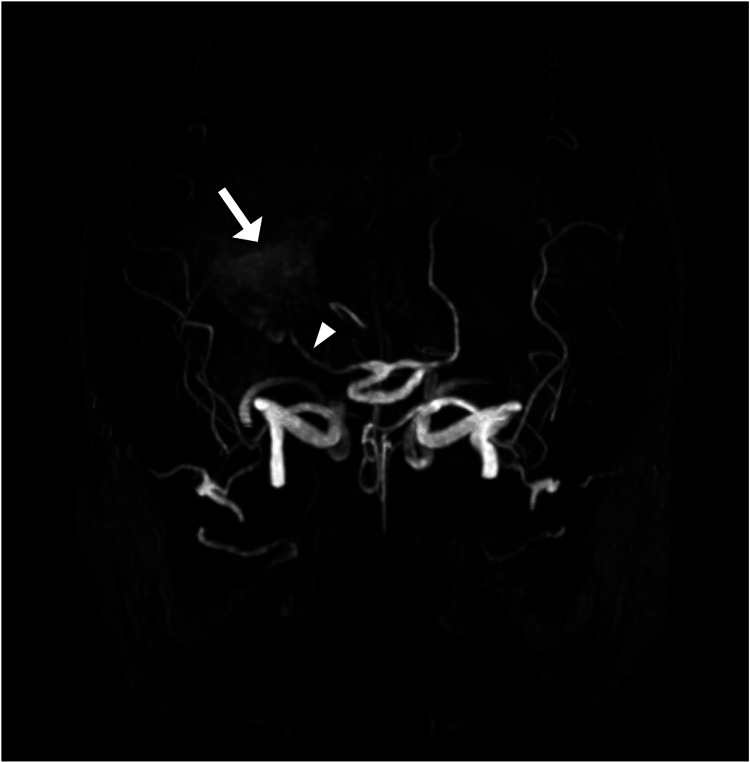
Three-dimensional volume-rendered magnetic resonance angiography reveals communication of the previously observed hemorrhage (denoted by a white arrow) and confirms it to be a large aneurysm in the posterior cerebral artery (denoted by a white arrowhead).

A magnetic resonance imaging of the brain demonstrated significant dilatation of the right posterior cerebral artery. Magnetic resonance angiography confirmed the presence of a large aneurysm (Figures 1, 2). These findings pointed towards the aneurysm as the likely cause of the patient's condition.

The patient was promptly admitted to the Neurology Department for further evaluation and management. Given the high risk associated with aneurysms and the patient's clinical condition, a multidisciplinary team, including neurosurgeons and interventional radiologists, was involved.

Endovascular coiling was considered the primary management option due to the aneurysm's size and location. After obtaining informed consent, the procedure was performed successfully, and the aneurysm was effectively coiled. The patient was closely monitored post-procedure for any complications.

During his hospitalization, the patient showed significant improvement in his symptoms. The severe headache resolved, and visual disturbances began to improve. His vital signs remained stable, and he did not develop any procedure-related complications.

## Discussion

Posterior cerebral artery aneurysms are relatively rare and distinct among intracranial aneurysms, constituting approximately 1% of all cases [1]. While these aneurysms are less common, their specific characteristics and management strategies warrant a closer look.

Previous cases and literature report that posterior cerebral artery aneurysms tend to be larger, with around 23% classified as large or giant [2]. In comparison to aneurysms in other anatomical locations, the prevalence of large aneurysms is notably higher in the posterior cerebral artery [2]. This unique characteristic underscores the importance of recognizing and understanding the distinct features of these aneurysms.

The posterior cerebral artery is responsible for supplying vital regions of the brain, including the temporal cortex, calcarine and occipital cortex, brainstem, and thalamus. Proximal P1 segment aneurysms can impede the oculomotor nerve, potentially leading to visual disturbances [3]. Our case aligns with this pattern, where the patient presented with right homonymous hemianopia, a specific and indicative visual field defect, emphasizing the importance of recognizing these clinical manifestations for early diagnosis.

Imaging techniques play a pivotal role in the early detection and characterization of posterior cerebral artery aneurysms. While noninvasive imaging studies such as computed tomography angiography and magnetic resonance angiography are valuable for routine screening and post-detection assessments, catheter angiography remains the gold standard for precisely defining aneurysms and their morphology [4]. This highlights the significance of various diagnostic tools in determining the precise location, size, and morphology of these aneurysms.

When considering the management of posterior cerebral artery aneurysms, it is imperative to weigh various factors, including the patient's age, aneurysm size, symptomatology, risk factors for rupture, and history of subarachnoid hemorrhage [5]. Our case underscores the critical need for prompt intervention due to the high risk associated with aneurysms and the potential for life-threatening complications. As our patient's condition warranted immediate attention, a multidisciplinary team was brought together, demonstrating the collaborative approach necessary for optimal patient care.

In the realm of treatment options, the case report highlights the success of endovascular coiling as a minimally invasive procedure. This approach, involving catheter-based interventions, is particularly advantageous for addressing challenging-to-reach aneurysms, such as those of the posterior cerebral artery [5]. As a less invasive alternative to surgical therapy, endovascular coiling provides an effective means of aneurysm occlusion, reducing the risks associated with more invasive surgical procedures.

## Conclusions

In conclusion, this case report underscores the clinical significance of posterior cerebral artery aneurysms, a relatively rare but potentially life-threatening vascular abnormality. Through the presented case, we have highlighted the distinct characteristics of these aneurysms, their diverse clinical manifestations, and the imperative role of prompt and multidisciplinary intervention. Imaging techniques play a pivotal role in the early diagnosis and characterization of aneurysms, allowing for tailored treatment strategies. While endovascular coiling has emerged as a minimally invasive and effective approach, careful consideration of patient-specific factors remains paramount in choosing the most suitable management strategy. This case serves as a reminder of the importance of collaborative care, informed decision-making, and comprehensive follow-up in ensuring optimal outcomes for patients with posterior cerebral artery aneurysms.
